# Assessment of T-dependent and T-independent immune responses in cattle using a B cell ELISPOT assay

**DOI:** 10.1186/1297-9716-43-68

**Published:** 2012-10-10

**Authors:** Clare FJ Grant, Eric A Lefevre, B Veronica Carr, Helen Prentice, Simon Gubbins, Andrew J Pollard, Catherine Charreyre, Bryan Charleston

**Affiliations:** 1 Institute for Animal Health, Compton Laboratory, High Street, Compton, Berkshire, RG20 7NN, United Kingdom; 2 Institute for Animal Health, Pirbright Laboratory, Ash Road, Pirbright, Surrey, GU24 0NF, United Kingdom; 3 Oxford Vaccine Group, Department of Paediatrics, University of Oxford, Oxford, OX3 9DU, United Kingdom; 4 Merial Animal Health, SAS, Avenue Tony Garnier, Lyon, 69007, France

## Abstract

Understanding the mechanisms that maintain protective antibody levels after immunisation is important for vaccine design. In this study, we have determined the kinetics of plasma and memory B cells detectable in the blood of cattle immunised with model T-dependent or T-independent antigens. Immunisation with the T-D antigen resulted in an expansion of TNP-specific plasma cells post-TNP primary and booster immunisations, which was associated with increased titres of TNP-specific IgG antibodies. Although no TNP-specific memory B cells were detected in the T-D group following the primary immunisation, we detected an increase in the number of TNP-specific memory B cells post-TNP boost. In contrast, no TNP-specific plasma or memory B cells were detected after primary or secondary immunisation with the T-I antigen. We then investigated if immunisation with a third party antigen (tetanus toxin fragment C, TTC) would result in a bystander stimulation and increase the number of TNP-specific plasma and memory B cells in the T-D and/or T-I group. TTC immunisation in the T-D group resulted in a small increase in the number of TNP-specific plasma cells post-TTC primary immunisation and boost, and in an increase in the number of TNP-specific memory B cells post-TTC boost. This bystander effect was not observed in the animals previously immunised with the T-I antigen. In conclusion, the present study characterised for the first time the B cell response in cattle to immunisation with T-D and T-I antigens and showed that bystander stimulation of an established T-D B cell memory response may occur in cattle.

## Introduction

Antibodies play a vital role in preventing viral infection and offer protection against subsequent re-challenge, providing protective antibody titres are maintained [1]. The maintenance of long-term protective antibodies following primary antigen exposure is provided by a combination of memory B cells and long-lived plasma cells, at least in mice [2]. On the basis of their size, nature and structure, antigens can induce T cell dependent (T-D) or T cell independent (T-I) immune responses [3]. We have previously shown that the B cell ELISPOT assay can be used to detect and enumerate antigen-specific plasma and memory B cells in cattle immunised with ovalbumin, a T-dependent (T-D) antigen [4]. However, there are currently no data available regarding the kinetics of these cells in the blood of cattle immunised with a T-independent (T-I) antigen.

Antigens that induce T-cell help to orchestrate a high affinity class-switched serological response are termed T-D antigens. During a T-D antigenic challenge, a small proportion of activated B cells differentiate into short-lived plasma cells within the T-cell regions of the secondary lymphoid organs and secrete low affinity antibodies for a short period of time [5]. The remaining activated B cells are recruited to the B cell follicles to form germinal centres, where the process of somatic hypermutation occurs (improving the B cell receptor affinity for their cognate antigen by 1 to 2 orders of magnitude) [6,7]; both long-lived plasma cells and memory B cells are generated and selected. Long-lived plasma cells migrate to specific niches within the bone marrow [8] and spleen [9], where they secrete high-affinity antibodies for prolonged periods [10]. In contrast, memory B cells continuously circulate without secreting antibodies.

Conversely, T-I antigens are able to initiate a serological response in the absence of T-cell help. There are two types of T-I antigens, type 1 are polyclonal B cell stimulants and type 2 are non-polyclonal stimulants. Type 2 T-I antigens possess highly organised, repeating structures that are able to activate naïve B cells in the absence of CD4^+^ T cell help, by cross linking multiple B cell receptors (BCRs) on the naïve B cell surface [11,12]. A second signal is required by the activated B cell to stimulate antibody production, either via TLR stimulation [3,13] or complement activation and CD21 stimulation [12]. However, whilst B cells can be activated by type 2 T-I antigens, development of long-term memory B cells to these antigens is limited, particularly in children under 2 years of age [14] and in neonatal mice [13]. It has been demonstrated that T-I antigens, such as polysaccharides, can be altered, via conjugation to a protein carrier, producing a T-D response, which results in the induction of sustained immunological memory [15,16].

Many pathogens contain both T-I and T-D antigens, virus capsids that have a repetitive/non-random structure, such as foot-and-mouth disease virus, FMDV, with antigenic epitopes spaced 5–10 nm apart) tend to preferentially generate a T-I immunological response [1,16]. Indeed, T cell depletion studies in cattle have shown that FMDV invokes a largely type 2 T-I response to structural proteins [17].

Upon re-exposure to a specific antigen [18] or upon polyclonal stimulation [10], memory B cells differentiate into plasma cells and secrete antibodies. Several mechanisms of polyclonal memory B cell stimulation have been previously described in mice and humans, including “bystander stimulation” from activated bystander CD4^+^ T-cells via cytokines [10], from microbial products via TLR stimulation (e.g. CpG DNA and LPS) [10], or from other B cell-activating molecules such as BAFF, APRIL and C4bp that are able to drive the differentiation of memory B cells into plasma cells in a T-independent manner [19]. This mechanism of “bystander stimulation”, thought to be an antigen-independent mechanism for the continuous replenishment of the plasma cell pool and thereby maintenance of protective antibody titres [10], has never been described in cattle.

In the present study, we assessed and compared the kinetics of plasma and memory B cells generated in cattle immunised with either a T-D antigen (the hapten 2,4,6-trinitrophenyl, TNP, conjugated to chicken gamma globulin, TNP-CGG) or type 2 T-I antigen (TNP conjugated to aminoethylcarboxymethyl-FICOLL, TNP-AECM-FICOLL). We also investigated whether a subsequent immunisation with a third party antigen exerted an effect upon the B cell response previously established to either the T-D or type 2 T-I antigen.

## Materials and methods

### Calves and immunisation protocols

Ten male 8-month-old conventionally reared Holstein-Friesian calves (*Bos Taurus,* Institute for Animal Health, Compton, UK) were split into two randomly selected groups of five. In the first group, each calf was subcutaneously immunised with 3 mg of the hapten 2,4,6-trinitrophenyl (TNP) conjugated to the T-D antigen chicken gamma globulin (TNP-CGG, Biosearch technologies, Novato, CA, USA) in QuilA adjuvant (Superfos Speciality Chemical a/s, Vedbaek, Denmark). In the second group, each calf was subcutaneously immunised with 3 mg of TNP conjugated to the T-I antigen aminoethylcarboxymethyl-FICOLL (TNP-AECM-FICOLL, Biosearch technologies, Novato, CA, USA) in QuilA adjuvant. All calves were boost-immunised 29-days later with the same immunising antigens, as described above (TNP-CGG in the T-D group and TNP-AECM-FICOLL in the T-I group). At day 134 post-primary immunisation (pi) all calves, from both groups, were subcutaneously immunised with 3 mg of tetanus toxin fragment C (TTC, PX’Therapeutics, Grenoble, France) in incomplete Freund’s adjuvant (IFA, Difco Laboratories, Detroit, MI, USA). Subsequently, all animals were boosted with TTC and IFA at day 197 pi. A group of control animals were given a primary and booster immunisation of adjuvant alone (including IFA). These animals showed no detectable antigen-specific plasma, memory B cells or IgG titres throughout the study. Heparinised peripheral blood and serum samples were taken from all animals at various time points following the immunisations. All experiments were approved by the Institute’s ethical review process and were in accordance with national guidelines on animal use.

### Bovine peripheral blood mononuclear cell (PBMC) isolation

Bovine PBMCs were isolated from the heparinised peripheral blood samples using density gradient centrifugation. The blood samples were diluted 1:2 using Dulbecco’s phosphate buffered saline (dPBS, Invitrogen), layered over Histopaque® 1.083 g/mL (Sigma-Aldrich) and centrifuged for 30 min at 1200 × *g*. The mononuclear cell layer was recovered after centrifugation and remaining red cells were lysed using ammonium chloride lysis buffer (155 mM NH_4_Cl, 10 mM KHCO_3_, 0.1 mM EDTA, pH7.2). After two further washing steps using dPBS, the PBMCs were enumerated using a Neubauer haemocytometer (Hawksley, Lancing, UK). A fraction of the enumerated fresh PBMCs were diluted to the required concentration and stored at 4°C until required for the detection of plasma cells.

### Memory B cell culture

An “ELISPOT medium” was used in all experiments aimed at quantifying plasma and memory B cells and consisted of RPMI 1640 with L-Glutamine and 25 mM Hepes (Invitrogen, Paisley, UK) supplemented with penicillin (100 units/mL), streptomycin (100 μg/mL), 1× non-essential amino acids (Sigma-Aldrich, Gillingham, UK), 1 mM sodium pyruvate and 15% heat-inactivated horse serum (Sigma-Aldrich).At the indicated time-points, some PBMCs were cultured as described below to induce the terminal differentiation of memory B cells into antibody secreting cells (ASC) prior to their detection by ELISPOT assay. Briefly, 2 × 10^7^ bovine PBMCs were cultured for 6 days in 20 mL of ELISPOT media (final PBMC concentration: 1 × 10^6^/mL) supplemented with 5 μg/mL pokeweed mitogen (PWM, Sigma-Aldrich), 10 μg/mL anti-bovine CD40 mAb (ILA158, kindly donated by ILRI, Kenya), 20 ng/mL recombinant human IL-2 (AMS Biotechnology Ltd, Abingdon, UK) and 7.3 units/mL bovine IL-10 (kindly donated by Dr G Entrican, Moredun Research Institute, Midlothian, UK). At the end of the culture period, the 6 day-cultured PBMC were recovered, washed twice in ELISPOT media, enumerated and stored on ice until being used for the detection of induced ASC by ELISPOT assay.

### Plasma and memory B cell quantification by ELISPOT

MultiScreen™–HA plates (Millipore, Watford, UK) were coated with either 0.25 μg/mL TNP-Ovalbumin (TNP-Ova), ^1^/_1000_ Chicken serum (containing CGG, Sigma-Aldrich) or 10 μg/mL TTC (PX’Therapeutics) in 0.1 M carbonate buffer (pH 9.6) for 2 h at 37°C. Plates were then washed 5 times in PBS and then blocked using 4% dried milk (Marvel, Premier Foods, St Albans, UK) in PBS. Following blocking, the plates were washed 5 times in PBS and stored at 4°C until required.

Fresh or 6 day-cultured PBMCs were suspended at 5 × 10^6^ cells/mL and 1:2 serial dilutions were performed in ELISPOT medium down to 1.5 × 10^5^ cells/mL. 100 μL/well of each suspension (in duplicate) was then incubated on the coated plates overnight at 37°C in a 5% CO_2_ incubator. Subsequently, the PBMCs were washed from the plates 5 times using PBS containing 0.05% Tween20 (Sigma-Aldrich). 100 μL per well of a ^1^/_1000_ dilution of sheep anti-bovine IgG conjugated to horseradish peroxidase (AbD Serotec, Kidlington, UK) was then added to the plates for 3 h at room temperature. Following the incubation step, the plates were again washed 5 times using PBS containing 0.05% Tween20. Detection was performed by the addition of 100 μL/well of 3-amino-9-ethylcarbonate substrate (AEC, Merck, Darmstadt, Germany) and subsequent incubation at room temperature for 1 h. The reaction was stopped by washing the plates with tap water and allowing them to dry overnight at room temperature.

Enumeration of the red coloured spots on the dried ELISPOT plates was performed using the automated AID ELIspot reader (Autoimmun Diagnostika GmbH, Strasbourg, Germany). Each individual “spot” was used as an indicator for an ASC. The ELISPOT results were manually validated for false positives and expressed as ASC number per 10^6^ PBMCs for plasma cells and induced-ASC number per 10^6^ cultured PBMCs for memory B cells (mean of duplicates). During the ELIspot data analysis any well with less than 2 spots was considered to be negative and were treated as having zero ASCs detected (minimum sensitivity: 4 ASCs per 1 × 10^6^ PBMCs). There was very little variance (< 17%) shown between the duplicate observations within each animal.

### Enzyme-linked immunoabsorbent assays (ELISA)

96-well plastic plates (BD biosciences, Oxford, UK) were coated for 1 h at room temperature with 100 μL/well of either 20 μg/mL TNP-OVA (Biosearch Technologies) or 10 μg/mL TTC (PX’Therapeutics) in carbonate buffer. Following incubation the plates were washed 5 times in PBS containing 1% Tween20 (Sigma-Aldrich). The plates were then blocked for 1 h using 100 μL/well of PBS containing 1% Tween20 and 4% dried milk (blocking buffer), followed by a washing step, as described above. Serum samples (in duplicate) were serially diluted 1:3 in blocking buffer starting at ^1^/_50_ dilution, and 100 μL per well of each diluted serum was added before incubating the plate for 1 h at room temperature. For determination of the plate background optical density (OD) values, some wells were incubated with blocking buffer alone. Following the incubation, the plates were washed and incubated for 1 h at room temperature after addition of 100 μL/well of ^1^/_100,000_ dilution of sheep anti-bovine IgG conjugated to horseradish peroxidase (AbD Serotec). Following washing, the plates were incubated for 10 min with 3,3’,5,5’-tetra-methyl benzidine (TMB, Sigma-Aldrich) substrate at room temperature. The reaction was stopped by the addition of 50 μL of 1.2 M H_2_SO_4_. The OD values at 450 nm and 690 nm were determined, for each well using a Biotek EL*x*808 absorbance plate reader (Biotek, Winooski, VT, USA). The antigen-specific antibody titres were calculated as follows: the log_10_ values of sample dilutions were plotted against the log_10_ values of sample OD and a regression analysis was performed on the linear part of the curve allowing the endpoint titre to be calculated using an OD of twice the background. Results were expressed as the mean of duplicate determinations.

### Statistical analysis

Data for the number of antigen-specific IgG ASCs (for plasma and memory B cells) were log + 1 transformed, then analysed using mixed linear models including time (as a factor) and vaccine (TNP-CGG or TNP-AECM-Ficoll), and an interaction between them as fixed effects and between animals as a random effect. Model selection proceeded by stepwise deletion of non-significant (*P* > 0.05) terms as judged by likelihood ratio tests. Data were considered significant if the calculated *P* values were < 0.05.

## Results

### Antibody titre kinetics following primary and booster immunisations with a T-D versus T-I antigen

**Figure 1:**
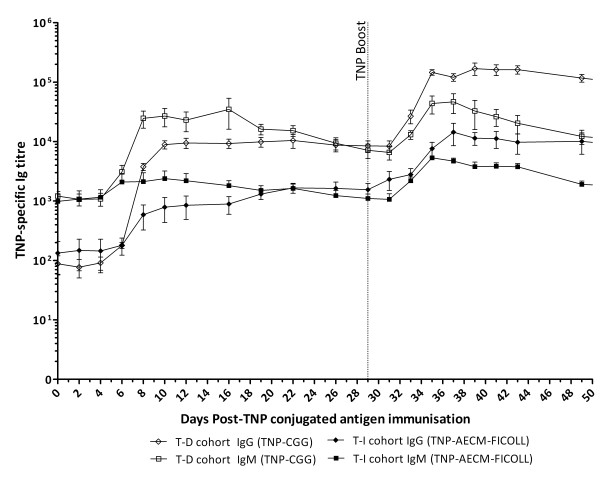
**Kinetics of TNP-specific Ig titres following primary and boost immunisations with TNP-conjugated antigens.** Five calves were subcutaneously immunised with 3 mg TNP-CGG or TNP-AECM-FICOLL in QuilA then boosted 29 days later with the same immunising antigens. The dotted vertical line indicates when the booster TNP-CGG/TNP-AECM-FICOLL immunisations were given (day 29 pi). Blood samples were taken at various time-points following these injections and TNP-specific IgG (diamond symbols) and IgM (square symbols) titres were determined by ELISA in calves from the T-D group (open symbols) or T-I group (filled symbols). Results are expressed as the mean of determinations from individual calves in each group ± SEM, *n* = 5.

The TNP-specific IgG titres in the T-D (TNP-CGG) and the T-I (TNP-AECM-FICOLL) groups began to increase from day 6–8 post-primary TNP immunisation (pi) and peaked at approximately day 10 pi for the T-D group (8905 ± 1417, *n* = 5) and approximately day 22 pi for the T-I group (1664 ± 314, *n =* 5) (Figure 1). Subsequently, the TNP-specific IgG titres in both groups also showed an increase starting from day 4 post-TNP boost that peaked at day 12 post-TNP boost (T-D group: 162 103 ± 33 339; T-I group: 11 226 ± 3573, day 41 pi, *n* = 5) (Figure 1). At most time-points, the T-I group TNP-specific IgG titres were over five times lower than the T-D group titres (Figure 1). The T-D group TNP-specific IgM titres showed an increase from day 6 pi, peaked at day 8 pi (24 635 ± 7854, *n* = 5) and remained elevated until day 16 pi (34 838 ± 18 730, *n* = 5) (Figure 1). Following boost immunisation, the IgM titres in the T-D group peaked at day 6 to 8 post-TNP boost (46 637 ± 17 044, day 37 pi, *n* = 5) before declining gradually (Figure 1). The T-I group also showed an increase in TNP-specific IgM titres, peaking 6 to 8 days after both the primary (2120 ± 867, day 8 pi, *n* = 5) and boost (4694 ± 578, day 37 pi, *n* = 5) TNP immunisations (Figure 1).

### Plasma cell and memory B cell kinetics following primary and booster immunisations with a T-D versus T-I antigen

**Figure 2:**
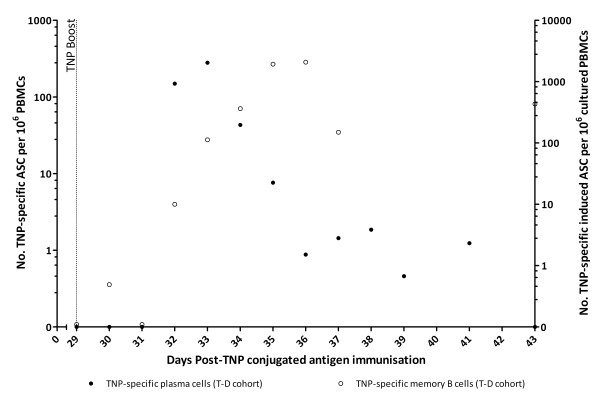
**TNP-specific plasma and memory B cell detection post-boost immunisation with TNP-CGG (T-D group).** Five calves were subcutaneously immunised with 3 mg TNP-CGG in QuilA then boosted 29 days later with the same immunising antigen. The dotted vertical line indicates when the booster TNP-CGG immunisation was given (day 29 pi). Blood samples were taken at various time-points following the immunisations. TNP-specific IgG, plasma cell (filled circles, left-axis) and memory B cell (open circles, right-axis) numbers were determined by B cell ELISPOT. Results were expressed as ASC per 10^6^ PBMC for plasma cells and induced ASC per 10^6^ cultured PBMC for memory B cells (mean of determinations from individual calves in each group, *n* = 5).

In the T-D group, there were no detectable TNP-specific plasma cells post primary immunisation. However, a burst of TNP-specific plasma cells was observed at days 3–5 post-TNP boost (280 ± 149 TNP-specific ASC per 10^6^ PBMC, day 33 pi, *n* = 5) (Figure 2). Although no TNP-specific memory B cells were detected in the T-D group following the primary immunisation (data not shown), TNP-specific memory B cells were detected from day 3 post-TNP boost, peaking at day 6–7 post-TNP boost (2080 ± 1218 TNP-specific induced ASC per 10^6^ cultured PBMCs, day 36 pi, *n* = 5) (Figure 2) and were still detectable at day 34 post-TNP boost (22 ± 3 TNP-specific induced ASC per 10^6^ cultured PBMCs, day 63 pi, *n* = 5, data not shown). At day 34 post-TNP boost, we also assessed the B cell memory response to CGG using chicken serum as a positive control within the assay. CGG-specific memory B cells were only detected in the T-D group and were approximately 5–6 fold more numerous than TNP-specific memory B cells (130 ± 40 CGG-specific induced ASC per 10^6^ cultured PBMCs, day 63 pi, *n* = 5, data not shown). In the T-I group, TNP- or CGG-specific plasma or memory B cells were not detected at any time-point following either the primary or booster TNP immunisation.

### Immunisation with TTC induces an increase in TTC-specific plasma and memory B cells

Each of the calves from both the T-D and T-I groups were immunised with a third party antigen (TTC) at day 134 pi and were re-immunised with TTC at day 197 pi.

**Figure 3:**
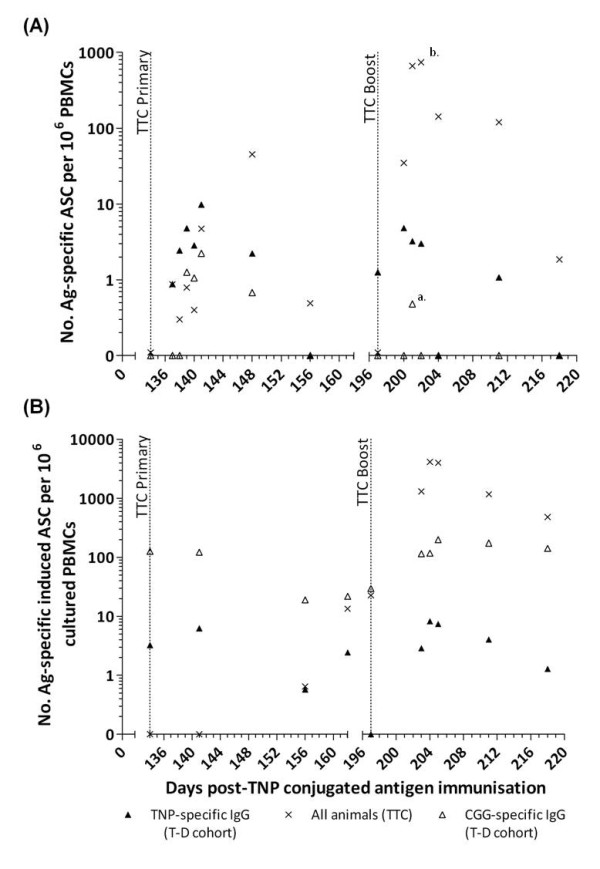
**Kinetics of antigen-specific IgG secreting plasma cells and memory B cells post-TTC immunisation.** At day 134 post-primary TNP-conjugated immunisation, all calves were subcutaneously immunised with 3 mg TTC in IFA then boosted 63 days later with the same immunising antigen. Blood samples were taken at various time-points following these immunisations. The time of primary and boost TTC immunisations are indicated by vertical dotted lines (day 134 and 197 pi, respectively). Antigen-specific IgG, plasma cell (**A**) and memory B cell (**B**) numbers were determined by B cell ELISPOT and are identified as follows: CGG-specific (open triangle), TTC-specific (crosses) and TNP-specific (closed triangle) IgG secreting ASCs. Results were expressed as ASC per 10^6^ PBMC for plasma cells and induced ASC per 10^6^ cultured PBMC for memory B cells (mean of determinations from individual calves in each group for the TNP- and CGG-specific responses, *n* = 5, and mean of determinations from all calves for the TTC-specific responses, *n* = 10). ^a^ = *p* < 0.05, ^b^ = *p* < 0.001.

A burst of TTC-specific IgG plasma cells was detected between day 7 to 14 post-primary TTC immunisation in all animals (45 ± 17 TTC-specific ASC per 10^6^ PBMCs, day 148 pi, *n* = 10) (Figure 3A). Following boost-immunisation with TTC at day 197 pi, TTC-specific plasma cells were detected from day 3 post-TTC boost and peaked at day 4 to 5 post-TTC boost (740 ± 126 TTC-specific ASC per 10^6^ PBMCs, day 202 pi, *n* = 10) (Figure 3A) (*p* < 0.001). A small burst of IgG TNP- (2 out of 5 calves) and CGG-(5 out of 5 calves) specific plasma cells was also observed in the T-D group at days 3 to 14 post-TTC primary immunisation (2 out of 5 calves showed 6 and 4 TNP-specific ASC per 10^6^ PBMCs, day 141 pi, *n =* 2 and 10 ± 6 CGG-specific ASC per 10^6^ PBMCs, day 141 pi, *n =* 5). Another small burst of CGG-specific plasma cells was observed in 3 out of 5 calves at days 3 to 5 post-TTC boost (3 out of 5 calves showed 4, 5 and 11 CGG-specific ASC per 10^6^ PBMCs, day 201 pi, *n =* 3) (*p* < 0.05) in the animals that had previously been immunised with the T-D antigen, TNP-CGG (Figure 3A). All of the animals demonstrated a steady increase in the number of TTC-specific memory B cells from day 28 post-TTC primary immunisation (13 ± 8 TTC-specific induced ASC per 10^6^ cultured PBMCs, day 162 pi, *n =* 10) (Figure 3B). The number of TTC-specific memory B cells increased significantly following the boost immunisation with TTC, peaking at days 6 to 8 post-TTC boost (4111 ± 495 TTC-specific induced ASC per 10^6^ cultured PBMCs, day 205 pi, *n =* 10) and then gradually declining (Figure 3B). TTC-specific memory B cells were still detectable in all animals at day 21 post-TTC boost (482 ± 110 TTC-specific induced ASC per 10^6^ PBMCs, day 218 pi, *n =* 10). There was also an increase in the number of TNP- and CGG-specific memory B cells in calves previously immunised with the T-D antigen at day 6–8 post-TTC boost (8 ± 5 TNP-specific induced ASC per 10^6^ cultured PBMCs and 200 ± 129 CGG-specific induced ASC per 10^6^ cultured PBMCs, day 205 pi, *n =* 5) (Figure 3B).

### Immunisation with TTC induces an increase in TTC-specific IgG titres in calves

**Figure 4:**
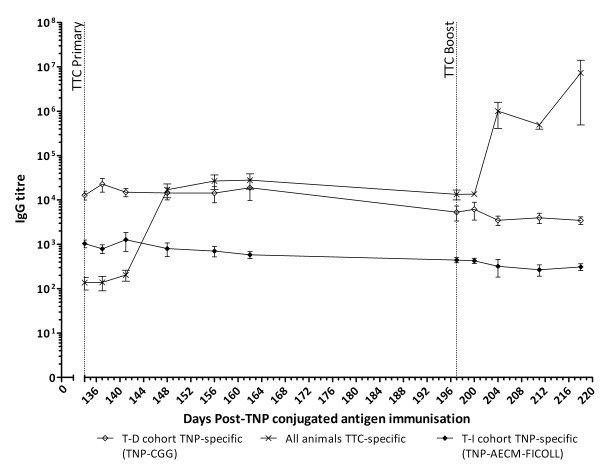
**Kinetics of TNP- and TTC-specific IgG titres following primary and boost TTC immunisations.** TNP-specific IgG titres in calves from the T-D group (open diamonds) or T-I group (filled diamonds) and TTC-specific IgG titres in all calves (cross symbols) were determined by ELISA. The dotted vertical lines indicate when the primary and boost TTC immunisations were administered (days 134 and 197 pi, respectively). Results are expressed as the mean of determinations from individual calves in each group for the TNP-specific responses (*n* = 5) and the mean of determinations from all calves for the TTC-specific responses (*n* = 10).

All animals demonstrated an increase in TTC-specific IgG titres from day 14 post-TTC primary immunisation (17 074 ± 5889, day 148 pi, *n* = 10) and day 7 post-TTC boost (1 004 210 ± 594 085, day 204 pi, *n* = 10) (Figure 4). There was a small, although not statistically significant, increase in TNP-specific IgG titres at day 3 post-TTC primary immunisation in the T-D group (22 794 ± 7762, day 137 pi, *n =* 5) as compared to day 0 post-TTC primary immunisation (12 868 ± 2913, day 134 pi, *n* = 5) (Figure 4). There was also a small increase in TNP-specific IgG titres at day 3 post-TTC boost in the T-D group (6186 ± 2675, day 200 pi, *n* = 5) as compared to day 0 post-TTC boost (5327 ± 1962, day 197 pi, *n =* 5). In the T-I group; TNP-specific IgG titres were not increased following either the TTC primary or boost immunisation (Figure 4).

## Discussion

This is the first time the magnitude and the kinetics of the T-D and T-I2 antigen specific B cell response has been described in cattle. Briefly, the response to the T-D antigen was characterised by an expansion of TNP-specific plasma cells peaking at day 7 post-TNP primary immunisation, which was associated with a subsequent increase in the titre of TNP-specific IgG antibodies. Following booster immunisation, a larger TNP-specific plasma cell burst was detected at day 3 to 5 post-TNP boost, and was similarly associated with a larger increase in TNP-specific IgG titres. The timing and magnitude of the T-D antigen-specific plasma cell bursts is in agreement with the findings of a previously published study, in which calves were immunised with another T-D model antigen, ovalbumin [4]. Moreover, a plasma cell burst has also been previously detected (with identical kinetics: 6 to 7 days post-primary immunisation) in humans following their immunisation with a number of different antigens, such as inactivated influenza virus [20], respiratory syncytial virus [21], serogroup C meningococcal polysaccharides [15] and tetanus toxoid [10]. The timing and magnitude of the secondary plasma cell burst and increased IgG response, i.e. a rapid and larger secondary response, is in keeping with the central dogma of immunological priming by a T-D antigen [15,22].

There was an increase in the number of TNP-specific memory B cells from day 3 post-TNP-CGG boost that peaked at day 6 to 7 post-TNP boost. TNP-specific and CGG-specific memory B cells were still detectable in the blood of calves from the T-D group at day 34 post-TNP boost. The timing and magnitude of this T-D antigen specific response, following booster immunisation, is in keeping with other previously published data [4]. Although no TNP-specific memory B cells were detected in the T-D group following the primary immunisation. Previous studies in both humans and cattle have shown low numbers of antigen-specific memory B cells following primary immunisation with a T-D antigen [4,15]. The rapid induction of TNP-specific plasma cells from day 3 post-TNP boost and the associated increase in TNP-specific IgG antibody titres observed in the T-D group suggests that a number of TNP-specific memory B cells were indeed generated after primary immunisation, but the quantities in blood were below the detection levels of our ELISPOT assay.

T-independent antigens are unable to prime individuals for a secondary immune response [15]. In this study the T-I2 group showed no detectable TNP-specific plasma or memory B cells, after either the primary or booster immunisation with TNP-AECM-FICOLL. Despite the lack of detectable antigen-specific plasma or memory B cells in the blood, there was a small increase in TNP-specific IgG titres in this group following primary and booster immunisations. It is likely that this increase in antibody titres results from the in situ generation of short-lived plasma cells, as previously described [5]. These findings are in keeping with Fink et al. who suggest that the absence of specific T cell help results in reduced germinal centre formation, which promotes an extra follicular plasma cell response, favouring short-lived plasma cell formation [22]. Kelly et al. have also shown that following primary immunisation with a T-I2 antigen, serogroup C meningococcal capsular polysaccharide, very low/undetectable numbers of antigen-specific memory B cells were generated [15]. The results from the present study shows that in cattle the kinetics and magnitude of the T-I2 antigen-specific plasma and memory B cell response is similar to that of the human and mouse.

Immunisation with the third party antigen (TTC) in this study resulted in similar TTC-specific plasma and memory B cell kinetics that have been previously described for other TD antigens [4,15,20,21]. Briefly, a burst of TTC-specific plasma cells was detected from day 7 to 14 post-TTC primary immunisation and from day 3 to 5 post-TTC boost in all animals. Interestingly, we found that the TTC-specific plasma cell bursts in the T-D group were also associated with a small increase in the number of TNP- and CGG-specific plasma cells post-TTC primary immunisation and/or boost. These results are consistent with those previously published by Bernasconi et al., which demonstrated a bystander polyclonal differentiation of memory B cells into plasma cells specific for various recall antigens (*Toxoplasma gondii* and measles virus) following the immunisation of humans with an unrelated T-D antigen (tetanus toxoid) [10].

There was a steady increase in the number of TTC-specific memory B cells post-TTC primary immunisation followed by at least a 14 fold increase in the number of these cells post-TTC boost. The T-D group also showed an increase in the number of TNP- and CGG-specific memory B cells only post-TTC boost (at day 6–8 post-TTC boost). Based on the timing of emergence of the TNP- and CGG memory B cells, it is unlikely that these memory B cells were generated from newly activated naïve B cells, but instead suggests that pre-existing TNP- and CGG-specific memory B cells were subjected to one (or more) round(s) of antigen-independent (bystander) proliferation within the T-cell regions of the draining lymph node. This bystander stimulation of T-D antigen-specific plasma and memory B cells may provide a mechanism for maintaining long term T-D antigen-specific antibody titres.

In conclusion, the present study showed that bystander stimulation of an established T-D B cell memory response may occur in cattle, providing a possible mechanism of maintaining protective antibody titres for long periods of time. Our findings also provided a better characterisation of the key differences between T-D and T-I immune responses in cattle. However, as highlighted by Fink et al., more studies are required to further elucidate the mechanisms of the T-D and T-I2 activation of antigen-specific plasma cells [22]. As demonstrated by this study and others [4,15,22], clearly the timing and magnitude of the antigen-specific plasma cell response is dependent on a number of factors, including the nature of the immunising antigen [22].

This knowledge will be particularly useful in elucidating the B cell response to the largely T-I antigen, FMDV [17]. Indeed as mentioned previously, most natural pathogens contain both T-I and T-D antigens, but viruses such as FMDV tend to preferentially generate a T-I immunological response [17]. FMDV is a highly contagious virus infecting cloven-hoofed animals, leading to skin erosions of the cutaneous mucosa. The virus has a significant global socio-economic impact, and the maintenance of FMDV-free status is critical for the free trade of animals and animal products [23]. One of the main methods of FMDV disease control and eradication is through vaccination [23]. The current FMDV vaccine only promotes short-term humoral immunity (presumably due to the essentially T-I nature of this virus), and regular repeat vaccinations are used to maintain protective IgG antibody titres [24], due to the lack of long-term immunological protection. Therefore, future studies including a more detailed assessment of the B cell response against FMDV in cattle could provide important data to underpin a better evaluation of novel candidate vaccines that induces a robust and sustained T-D rather than a T-I response.

## Abbreviations

ASC: Antibody secreting cell; T-I: T-independent; T-D: T-dependent.

## Competing interests

The authors declare that they have no competing interests.

## Authors’ contributions

CFJG carried out the majority of the immunoassays performed in this study, the data interpretation and drafted the manuscript. EAL participated in the design of the study, carried out some of the immunoassays and revised the manuscript. BVC performed some of the immunoassays. HP participated in the design of the study and study coordination. SG performed the statistical analysis. AJP participated in the design of the study, data interpretation and manuscript revision. CC participated in the study design and coordination. BC conceived the study and revised the manuscript. All authors have read and approved the final manuscript.
